# Performance of Ultrasensitive Polymerase Chain Reaction Testing for JC Polyomavirus in Cerebrospinal Fluid Compared with Pathological Diagnosis of Progressive Multifocal Leukoencephalopathy

**DOI:** 10.3390/v16121950

**Published:** 2024-12-19

**Authors:** Kenta Takahashi, Kazuo Nakamichi, Yuko Sato, Harutaka Katano, Hideki Hasegawa, Masayuki Saijo, Tadaki Suzuki

**Affiliations:** 1 Department of Pathology, National Institute of Infectious Diseases, Shinjuku-ku, Tokyo 162-8640, Japan; tkenta@niid.go.jp (K.T.); kiyonaga@niid.go.jp (Y.S.); katano@niid.go.jp (H.K.); hasegawa@niid.go.jp (H.H.); 2 Department of Virology 1, National Institute of Infectious Diseases, Shinjuku-ku, Tokyo 162-8640, Japan; masayuki.saijo@doc.city.sapporo.jp; 3 Research Center for Influenza and Respiratory Viruses, National Institute of Infectious Diseases, Musashimurayama-shi 208-0011, Tokyo, Japan; 4 Public Health Office, Health and Welfare Bureau, City of Sapporo, Sapporo-shi 060-0042, Hokkaido, Japan

**Keywords:** brain biopsy, cerebrospinal fluid, JC polyomavirus, progressive multifocal leukoencephalopathy, real-time PCR, ultrasensitive

## Abstract

Progressive multifocal leukoencephalopathy (PML) is a demyelinating disease caused by the JC polyomavirus (JCPyV). Based on the clinical criteria, PML is diagnosed via polymerase chain reaction (PCR) detection of JCPyV DNA in cerebrospinal fluid (CSF) in combination with neurological and imaging findings. Although the utility of CSF JCPyV testing using ultrasensitive PCR assays has been suggested, its potential requires further evaluation. This study retrospectively analyzed the detection performance of ultrasensitive PCR for CSF JCPyV in patients who underwent brain tissue examination based on the pathological diagnostic criteria for PML. Of the 110 patients with pathologically confirmed definite PML or not PML, standard and ultrasensitive CSF testing was performed for 36 and 74 patients, respectively. The sensitivity of ultrasensitive CSF JCPyV testing of the initial specimens was 85%. With the addition of the follow-up testing, this figure increased to 95%. The specificity and false-positive rate of ultrasensitive CSF JCPyV testing, including follow-up, were 100% and 0%, respectively. No statistically significant correlation was observed between CSF and brain JCPyV levels. The results of this study demonstrate the high sensitivity and accuracy of ultrasensitive CSF JCPyV testing and provide essential information for the clinical diagnosis of PML.

## 1. Introduction

Progressive multifocal leukoencephalopathy (PML) is a demyelinating disease that primarily occurs in immunosuppressed patients and is often fatal [1,2,3]. In addition to patients with hematopoietic malignancies, acquired immunodeficiency syndrome (AIDS), and autoimmune diseases, PML has also recently been reported in individuals receiving antibody drugs or immunomodulatory therapies [4,5,6,7,8,9]. The etiological agent of PML is the JC polyomavirus (JCPyV, also known as JC virus or JCV), which belongs to the *Polyomaviridae* family and has a circular double-stranded DNA genome [10,11]. Lytic infection of oligodendrocytes by JCPyV results in demyelination of the brain [10,11,12].

PML is diagnosed based on two criteria: clinical features and pathological findings [13]. Clinical features are determined based on clinical manifestations, magnetic resonance imaging patterns, and polymerase chain reaction (PCR) detection of JCPyV DNA in cerebrospinal fluid (CSF). Pathological findings require evidence of histopathological features and the detection of JCPyV in brain tissue using immunohistochemistry (IHC), electron microscopy, or PCR. The most certain diagnosis for each of these criteria is defined as “definite PML”. Cases in which PML is ruled out are referred to as “not PML”.

Although the accuracy of the pathological examination of brain biopsies in diagnosing PML is high [14,15], given the invasiveness of surgery, initial PCR testing for CSF JCPyV is the preferred clinical approach. In patients with PML, the sensitivity and specificity of PCR testing for JCPyV in CSF have been reported to be 72–92% and 92–100%, respectively [16,17,18]. The sensitivity of CSF testing for JCPyV varies by facility, and the detection limit for real-time PCR in many commercial laboratories (hereafter, “standard PCR assay”) is approximately 200 viral genome copies per milliliter of sample [19]. The sensitivity of PCR detection of JCPyV in CSF is influenced by the type of underlying disease or its treatment. In a study that limited its cohort to patients with AIDS-related PML in the era of antiretroviral therapy, the sensitivity of this test was reported to be as low as 58% [20]. There are several reports of patients who tested negative for JCPyV in their CSF and were subsequently diagnosed with PML via a brain biopsy [21,22,23,24].

To improve the detection of JCPyV in CSF, virologists have developed a highly sensitive real-time PCR test that utilizes enriched DNA and/or viral particles in the sample (hereafter, “ultrasensitive PCR assay”) [25,26,27]. An ultrasensitive PCR assay can quantitatively detect JCPyV DNA at a lower limit of 10–20 copies/mL in CSF, suggesting its utility in diagnosing PML [4,13,19]. However, the performance of ultrasensitive PCR testing for CSF JCPyV compared with the pathological examination of brain tissue is not well understood. The sensitivity and specificity of CSF JCPyV testing reported in previous studies were primarily based on the results of qualitative analysis using conventional (not real-time) or nested PCR for patients with AIDS-related PML [13,16,17,18,20]. The precise relationship between the JCPyV copy number in CSF and brain tissue based on quantitative PCR remains unclear. Moreover, the diagnosis of PML due to other predisposing factors, such as disease-modifying drugs for multiple sclerosis (MS), employs ultrasensitive CSF testing; however, a brain biopsy is seldom conducted [19,28]. Collecting and integrating the results of ultrasensitive CSF testing for JCPyV and brain pathologies from different medical institutions is challenging.

In Japan, PCR testing for JCPyV in CSF at the National Institute of Infectious Diseases (NIID) has been ongoing since 2007 as diagnostic support and nationwide surveillance for PML [4,29]. A search of the subject database for CSF JCPyV testing revealed that more than 100 patients underwent brain tissue examinations to diagnose PML at the pathology department of the same institution. This study retrospectively analyzed the detection performance of an ultrasensitive PCR assay for CSF JCPyV in patients who underwent pathological diagnosis of the brain tissue for PML.

## 2. Materials and Methods

### 2.1. Subjects and Clinical Data

This study was approved by the Medical Research Ethics Committee for the Use of Human Subjects at the NIID (approval nos. 822, 1689, and 1748). The study was conducted in accordance with the ethical standards of the Declaration of Helsinki. Written informed consent was obtained from patients or their authorized representatives. Specimens from patients with suspected PML were sent by medical institutions to the NIID, and the CSF and brain tissues were analyzed in the Departments of Virology 1 and Pathology, respectively. Patient information, including their age, sex, and underlying medical conditions, was anonymized by the treating physician and obtained at the time of each test.

### 2.2. PCR Testing for JCPyV DNA in CSF

A total of 2418 patients with suspected PML were subjected to real-time PCR testing for CSF JCPyV, of whom 469 tested positive between July 2007 and May 2024. This period was selected as the study period. Of these, a standard PCR assay capable of detecting at least 200 copies/mL of JCPyV DNA in CSF was performed in 1274 patients [29,30,31]. In April 2016, an ultrasensitive PCR assay for CSF JCPyV with a lower detection limit of 10 or 20 copies/mL was introduced and employed to diagnose 1144 individuals [4,27]. Since 2007, several PCR assays for CSF JCPyV have been implemented at the NIID due to improved detection protocols, equipment updates, and international standardization. The quantitative performance of each assay has been confirmed using the complete JCPyV genome as a reference. Details of these methods, including those for PCR testing of brain tissue, are summarized in the Supplementary Materials (Datasets S1 and S2). In 2013–2016, the QIAamp Viral RNA Mini Kit (Qiagen) was used to extract nucleic acid from a small volume of CSF sample without protease treatment for the standard PCR assay (Dataset S1). This kit can rapidly extract both DNA and RNA from liquid samples according to the manufacturer’s protocol, as described previously [31]. The primary distinction between standard and ultrasensitive PCR assays for CSF JCPyV used in this study lies not in the amplification efficiency, such as primers, probes, premix, temperature cycling, or reagents (Dataset S2), but in the rate of nucleic acid enrichment, including viral DNA, which is extracted from the CSF sample [25,26,27]. The ultrasensitive PCR assay uses the QIAamp MinElute Virus Spin Kit (Qiagen) with modifications to the manufacturer’s protocol for the extraction of nucleic acid from larger volumes of CSF (Dataset S1). The current (2024) ultrasensitive PCR test for CSF JCPyV at the NIID utilizes a previously reported nucleic acid extraction protocol with manual handling [27], PCR primers and probe [25], and the LightCycler 96 platform [4].

Key considerations for performing ultrasensitive CSF JCPyV testing include managing the increased risk of false positives due to specimen contamination in laboratories that handle a variety of specimens, especially urine, because archetype JCPyV persists in many individuals. We have a dedicated laboratory for CSF JCPyV testing in the NIID. When the JCPyV genome is detected in CSF, it is often necessary to analyze mutations in the prototype virus characteristic of PML [26,32,33,34,35]. Furthermore, ultrasensitive CSF JCPyV testing requires skilled manual concentration of high-purity nucleic acids to maintain PCR efficiency. Finally, it is crucial to validate that ultrasensitive JCPyV testing is effective across a large number of CSF samples from patients with and without PML [4,27,36]. Our verification testing at the NIID has identified many cases of false-positive or false-negative CSF JCPyV tests in other local laboratories in Japan, likely due to inadequate specialized skills or clinical validation of the assays (submission in preparation). For this reason, we recommend that ultrasensitive CSF JCPyV testing be conducted by experts in research institutions after clinical validation of the assay based on patient data.

### 2.3. Pathological Examination of Brain Tissues

Formalin-fixed and paraffin-embedded (FFPE) brain tissues from patients with suspected PML were histopathologically evaluated using hematoxylin and eosin (HE) staining. IHC was conducted using antibodies against JCPyV VP1, VP2/3, and/or agnoprotein, as previously described [37,38,39]. To detect JCPyV in the brain tissue, a real-time PCR assay was performed on DNA extracts from frozen and/or FFPE samples, as previously described [39,40] (Supplementary Datasets S1 and S2). The number of JCPyV DNA copies per cell was calculated based on the housekeeping gene beta-actin [41,42]. At instances where both frozen and FFPE samples from the same patient were subjected to PCR testing, the results of the FFPE section were given precedence because they should be more closely related to the evaluation of morphological characteristics. Diagnoses of pathologically definite PML and not PML were made through morphological evaluation with IHC and/or tissue real-time PCR in accordance with the PML diagnostic criteria [13].

### 2.4. Data Analysis

**Figure 1:**
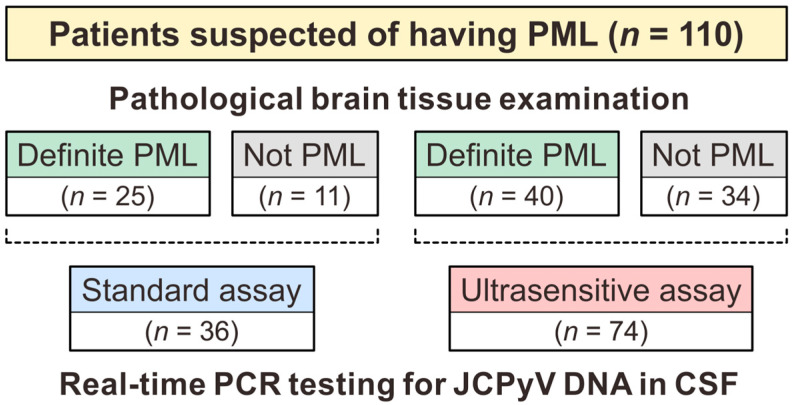
Schematic diagram of study subjects. At the National Institute of Infectious Diseases in Japan, 110 patients with suspected PML underwent examination of both their CSF and brain tissue. Brain tissue samples were examined according to the pathological diagnostic criteria for PML. The number of patients diagnosed with definite PML and not PML are shown. For CSF JCPyV testing, two patient groups underwent either standard or ultrasensitive real-time PCR assays. PML, progressive multifocal leukoencephalopathy; PCR, polymerase chain reaction; JCPyV, JC polyomavirus; CSF, cerebrospinal fluid.

In cases where both CSF and brain tissue examinations are performed for JCPyV, brief information about their implementation is routinely shared with the Departments of Virology 1 and Pathology of the NIID. By cross-referencing the database of subjects who underwent CSF JCPyV testing with records of brain tissue examinations, 110 applicable patients out of 2418 suspected PML cases were identified. The composition of the study population is shown in Figure 1. In all cases, both CSF and brain tissue samples were collected at intervals of no more than 6 months. Among the individuals with suspected PML who underwent standard CSF JCPyV testing (*n* = 36), 25 and 11 were diagnosed with definite PML and not PML, respectively, via pathological examination of the brain tissue. Similarly, 40 and 34 patients who underwent ultrasensitive CSF JCPyV testing, with a lower detection limit of 20 copies/mL, were pathologically diagnosed with definite PML and not PML, respectively. Although ultrasensitive PCR can detect JCPyV to a lower limit of 10 copies/mL by concentrating virus particles in the CSF using ultrafiltration [27], the patients in this study were tested without ultrafiltration. When a very weak signal is observed during this routine ultrasensitive PCR test (>20 copies/mL), we perform the method that can detect JCPyV to a lower limit of 10 copies/mL by concentrating virus particles in the CSF using ultrafiltration [27]. However, no such findings were observed in the patients in this study, and the ultrasensitive PCR testing with an ultrafiltration procedure was not performed.

### 2.5. Statistics

The proportions of patients with clinical features among those pathologically diagnosed with definite PML and not PML were compared using the two-tailed Fisher’s exact test. The copy number of JCPyV DNA in each group was analyzed using the Mann–Whitney U-test. The correlation between JCPyV DNA loads in the CSF and brain tissue samples was assessed using Spearman’s rank correlation coefficient. All *p*-values < 0.05 were considered statistically significant.

## 3. Results

### 3.1. Clinical Characteristics of the Study Population

The results of the pathological examinations of brain tissues from patients with suspected PML at a national laboratory in Japan are yet to be published in a consolidated manner. The clinical characteristics of the patients in this study, including the pathological diagnosis of PML, are summarized and categorized in Table 1. Based on the pathological examination of their brain tissues, 65 and 45 of the 110 patients were diagnosed with definite PML and not PML, respectively. The mean age of the patients in the definite PML and not PML groups was approximately 60 years, with a similar male-to-female ratio. The patients in both groups had various underlying diseases, and CSF testing for JCPyV was often performed before brain tissue examination. In the definite PML group, in a small number of cases, the JCPyV antigen was not detected in the brain tissue using IHC, or this test was not used. However, all patients tested positive for JCPyV using PCR testing of FFPE or frozen tissues. Statistical analyses indicated that the not PML group exhibited a higher prevalence of patients with MS or without clinically evident underlying disease than the definite PML group (*p* = 0.025 and 0.009, respectively). Nevertheless, no statistically significant differences were observed in the remaining subcategories between the two groups, except for positive IHC and JCPyV PCR rates (*p* < 0.001 for each). These data indicate that the study participants pathologically diagnosed with definite PML and not PML had similar clinical features and examination processes.

### 3.2. Detection Performance of Real-Time PCR Testing for JCPyV in CSF

The overall performance of the standard and ultrasensitive PCR assays for CSF JCPyV testing was evaluated in patients with a pathological diagnosis of PML in the brain tissue (Table 2). When JCPyV testing was performed only on the CSF specimens collected at the time of initial testing, the sensitivities (true-positive rates) of the standard and ultrasensitive PCR assays were 40% and 85%, respectively. In the definite PML group, the positivity rate of the ultrasensitive PCR assay was significantly higher than that of the standard PCR assay (*p* < 0.001). Some patients underwent additional ultrasensitive PCR testing for CSF JCPyV (once for three patients; twice for one patient), and the sensitivity of this assay, including the results from follow-up testing, increased to 95%. No statistically significant differences were observed in the CSF JCPyV positivity rates before and after the collection of brain tissues, regardless of whether standard or ultrasensitive PCR assays were used. The false-positive rates and positive predictive values for the two PCR assays were 0% and 100%, respectively. While the specificities (true-negative rates) of both PCR assays were 100% in initial CSF testing, the false-negative rates for the standard and ultrasensitive methods were 60% and 15%, respectively. The negative predictive values for the standard and ultrasensitive methods in initial CSF testing were 42% and 85%, respectively. When follow-up CSF testing was performed with the ultrasensitive PCR assay, the false-negative rate dropped to 5%, and the negative predictive value reached 94%. These results demonstrate that the ultrasensitive PCR assay has a high detection performance for JCPyV in CSF without generating false-positive reactions.

### 3.3. JCPyV Copy Levels in the CSF and Brain Tissue of Patients with PML

**Figure 2:**
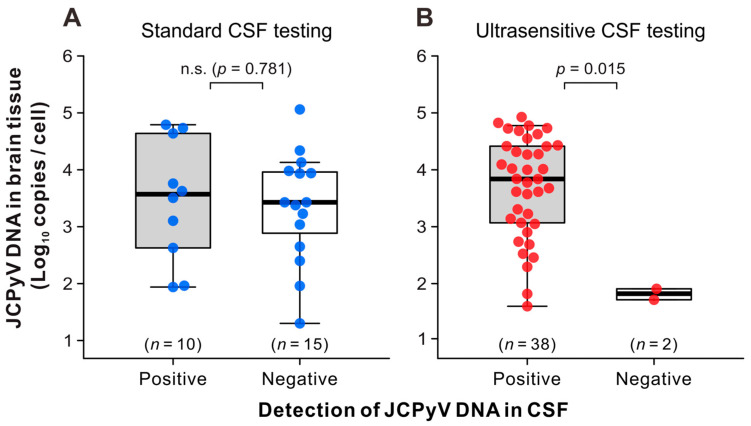
JCPyV DNA levels in the brain tissues of patients diagnosed with pathologically definite PML. The copy numbers of JCPyV genomic DNA in the brain tissues were measured using real-time PCR, as described in the main text, and are presented using combinations of bee swarm and box-and-whisker plots. The data were divided into groups in which real-time PCR testing for CSF JCPyV was performed using standard (**A**) and ultrasensitive (**B**) assays. In each panel, the JCPyV copies per cell in the brain tissues of patients with positive (**left**) and negative (below the detection limit) (**right**) results in CSF JCPyV testing are shown. The vertical axes show the logarithms of the JCPyV loads. The thick horizontal line within each box represents the median, and the lower and upper limits represent the 25th and 75th percentiles, respectively. Vertical whiskers extend to 1.5 times the interquartile range of the data. CSF, cerebrospinal fluid; n.s., not significant; JCPyV, JC polyomavirus.

**Figure 3:**
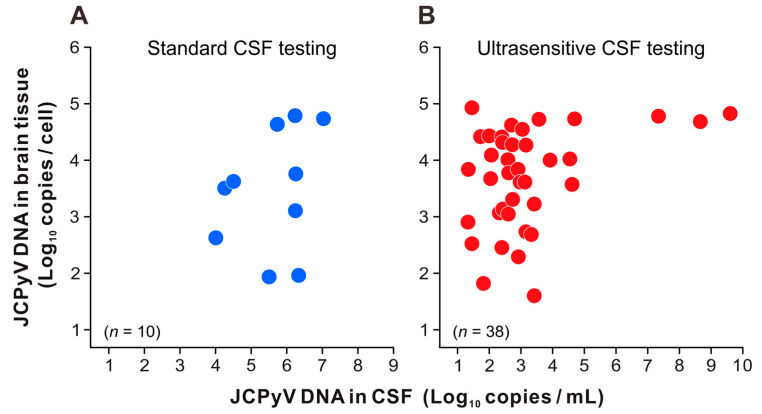
Comparison of viral DNA levels in patients positive for JCPyV in both the CSF and brain tissues. JCPyV DNA levels in the CSF (copies/mL) and brain tissues (copies/cell) were quantified using PCR, as described in the main text. The data were divided into groups in which real-time PCR testing for CSF JCPyV was performed using standard (**A**) and ultrasensitive (**B**) assays. In each panel, the vertical and horizontal axes represent the logarithmic values of JCPyV DNA loads in brain tissues and CSF, respectively. CSF, cerebrospinal fluid; JCPyV, JC polyomavirus.

The amount of JCPyV DNA in the brain tissue was compared between the two groups of patients with pathologically definite PML who tested positive or negative for JCPyV in their CSF. In patients with PML who underwent standard PCR testing for CSF JCPyV, viral DNA loads in the brain tissue were distributed between approximately 10 and 10^5^ copies per cell (Figure 2A). There were no statistically significant differences in the number of JCPyV copies in the brain tissue between the two groups that tested positive or negative in standard CSF testing. Similarly, the JCPyV DNA levels were widely distributed in the brains of patients with PML who tested positive for CSF JCPyV using ultrasensitive PCR testing (Figure 2B, left). However, JCPyV copies in the brain tissues of patients with PML who tested negative on ultrasensitive CSF testing were significantly lower (*p* = 0.015) (Figure 2B, right). Next, the levels of JCPyV DNA detected in the brain tissue and CSF of each patient with PML were plotted and compared. Compared to standard CSF testing (Figure 3A), ultrasensitive CSF testing detected JCPyV in the range of 20–10^3^ copies/mL in many cases (Figure 3B). The number of JCPyV copies in the CSF did not demonstrate a statistically significant discrepancy between patient groups in which the brain tissue was obtained prior to or following CSF testing. Moreover, no statistically significant correlation was identified between the CSF and brain JCPyV DNA levels in either patient group (Figure 3A,B). These results suggest that the high and low copy numbers of JCPyV in the CSF and brain tissues of patients with PML do not necessarily follow the same trend.

## 4. Discussion

This study evaluated the detection performance of an ultrasensitive PCR assay for JCPyV DNA in CSF samples, with a particular focus on patients who underwent pathological examination of brain tissues for the diagnosis of PML. Previous reports on the performance of PCR testing for CSF JCPyV have included data using older PCR technologies or the data based on either clinical or pathological diagnostic criteria for PML. We believe that the major significance of this study is that it analyzed the brain tissue and CSF findings of over 100 patients with suspected PML at a single institution and updated the integrated information not only on the performance of the ultrasensitive CSF JCPyV testing but also on the two diagnostic criteria for PML.

The sensitivity of the ultrasensitive PCR assay for CSF JCPyV was 85% at the time of initial testing and increased to 95% after the addition of follow-up testing. These data indicate that the sensitivity of the ultrasensitive PCR assay for CSF JCPyV is indeed superior to that of the standard real-time PCR assay, as well as to that of conventional or nested PCR assays reported previously [16,17,18]. In a previous study of a large number of MS cases, JCPyV was detected during ultrasensitive PCR testing of CSF samples from patients not clinically diagnosed with PML [36]. However, in this study, the positive predictive value of ultrasensitive CSF testing was 100% and no false positives were observed in patients pathologically diagnosed with “not PML”. Differences in the underlying diseases, number of patients, onset of PML symptoms, and PML diagnostic criteria (clinical or pathological) were observed between the two studies. In pathologically confirmed cases, the frequency of JCPyV detection in the CSF of patients without PML is extremely low.

The specificity of CSF JCPyV testing using the standard and ultrasensitive methods was 100% for both; however, the standard method had a high false-negative rate. When follow-up testing was added, the false-negative rate of the ultrasensitive PCR assay improved by 5%, and its negative predictive value reached 94%. However, as reported in previous studies [21,22,23,24], JCPyV was not detected in the CSF samples of 2 of the 40 patients despite a pathological diagnosis of definite PML. In these patients, the symptoms, imaging findings, and predisposing conditions (rheumatoid arthritis and multiple myeloma, respectively) were consistent with PML. However, because JCPyV was not detected in their CSF, it was difficult to confirm definite PML based on clinical diagnostic criteria alone. Additionally, although brain tissue examinations offer various insights into PML lesions, CSF PCR testing is limited to detecting and quantifying the JCPyV genome. There were some cases in this study where extremely low levels of JCPyV were detected in the CSF, and a brain biopsy was performed to confirm the diagnosis of PML. Therefore, a brain biopsy is essential and should be carefully considered, especially in cases with repeated negative CSF results and extensive clinical investigations.

The findings of this study indicate that the viral loads of JCPyV in CSF and brain tissues may not be directly proportional. Specifically, it is conceivable that JCPyV actively replicates or accumulates in infected cells within PML lesions, even when CSF JCPyV is negative or positive at trace levels. The discrepancy in JCPyV DNA levels in the CSF and brain tissue is thought to result from several factors, including the timing of specimen collection, disease progression, and CSF dynamics. A positive correlation between the total PML lesion volume and CSF JCPyV copy number has been reported in patients treated with natalizumab [43]. The size of the brain lesions and/or their proximity to the CSF cavity may also influence the quantitative results. Even if JCPyV is not detected in the CSF, it may replicate in local lesions. Brain biopsies can detect JCPyV with a high degree of certainty if sampling is successful. Therefore, brain biopsy specimen selection is extremely important, and it is desirable to collect specimens that are suitable for viral testing. To this end, it is necessary to determine which areas of the brain lesions contain larger amounts of viruses.

Among the patients diagnosed with definite PML in this study, there were cases in which brain tissue was collected before CSF sampling because of a suspected brain tumor or lymphoma. Surgical procedures may cause JCPyV to leak from the PML lesion into the CSF, resulting in a high detection rate of the virus in the CSF. However, the results of both the standard and ultrasensitive PCR assays provided no evidence that the prior collection of brain tissue affected the positivity rate or copy number of JCPyV in the CSF. In most definite PML cases where brain tissue was obtained before CSF testing, there was an interval of more than 2 weeks between the brain biopsy and CSF sampling. It is postulated that JCPyV that leaked from the brain lesion into the CSF may have been eliminated via circulation during this period. Further analysis is needed to determine the effect of the timing of CSF sampling and the brain biopsy on the PCR results for CSF JCPyV.

A potential limitation of this study is the possibility of bias in the population of this study owing to the existence of two diagnostic criteria for PML: clinical and pathological. Many patients with definite PML are clinically diagnosed without a pathological brain examination in cases where the diagnostic algorithm is followed [13] or brain lesions are difficult to obtain [44]. However, to evaluate the performance of CSF JCPyV testing more rigorously, it is necessary to exclude definite PML cases without pathological confirmation from the study design. This may explain why the sensitivity of the standard PCR assay for CSF JCPyV was lower in this study than that previously reported [16,17,18,20]. If definite PML cases diagnosed using clinical criteria alone were included in the study population, the practical sensitivity of CSF JCPyV testing would have been higher than the results of this study.

## 5. Conclusions

The present study retrospectively analyzed the detection performance of an ultrasensitive PCR assay for JCPyV in CSF samples from patients who underwent pathological examination of their brain tissue for the diagnosis of PML. The results of this study demonstrate the high sensitivity and accuracy of ultrasensitive CSF JCPyV testing and provide essential information for the clinical diagnosis of PML.

## Figures and Tables

**Table 1:** Clinical characteristics of patients pathologically diagnosed with definite PML and not PML.

Category	Subcategory	All		Definite PML		Not PML	
		*n* = 110		*n* = 65		*n* = 45	
Age	n.a.	59.9 ± 14.1		60.3 ± 13.3		59.2 ± 15.3	
Sex	Male	60	(55)	34	(52)	26	(58)
	Female	50	(45)	31	(48)	19	(42)
Underlying diseases ^a^	Hematopoietic malignancy	30	(27)	20	(31)	10	(22)
	Autoimmune diseases	24	(22)	18	(28)	6	(13)
	AIDS	14	(13)	9	(14)	5	(11)
	Solid organ cancer	10	(9)	5	(8)	5	(11)
	Solid organ transplantation	8	(7)	5	(8)	3	(7)
	Renal dysfunction	4	(4)	3	(5)	1	(2)
	Sarcoidosis	3	(3)	3	(5)	0	(0)
	Congenital immunodeficiency	2	(2)	2	(3)	0	(0)
	Acquired immunodeficiency ^b^	2	(2)	2	(3)	0	(0)
	Hepatic dysfunction	2	(2)	2	(3)	0	(0)
	Multiple sclerosis	4	(4)	0	(0)	4	(9)
	Not obvious	15	(14)	4	(6)	11	(24)
	Uncertain	1	(1)	0	(0)	1	(2)
CSF sampling	Before brain tissue examination	84	(76)	49	(75)	35	(78)
	After brain tissue examination	26	(24)	16	(25)	10	(22)
Brain tissue sampling	Biopsy ^c^	99	(90)	56	(86)	43	(96)
	Autopsy	11	(10)	9	(14)	2	(4)
Immunohistochemistry	Positive	59	(54)	59	(91)	0	(0)
	Negative	38	(35)	5	(8)	33	(73)
	Not performed	13	(12)	1	(2)	12	(27)
Brain DNA extraction	FFPE sample ^d^	78	(71)	51	(78)	27	(60)
	Frozen sample	32	(29)	14	(22)	18	(40)
JCPyV PCR of brain tissue	Positive	65	(59)	65	(100)	0	(0)
	Negative	45	(41)	0	(0)	45	(100)

Data are presented as numbers and percentages (%) of patients who underwent pathological examination of their brain tissues, except for age, which is presented as the mean and standard deviation. PML, progressive multifocal leukoencephalopathy; n.a., not applicable; AIDS, acquired immunodeficiency syndrome; CSF, cerebrospinal fluid; FFPE, formalin fixed and paraffin embedded; JCPyV, JC polyomavirus. ^a^ Patients with complications and/or comorbidities were included in multiple underlying disease subcategories. ^b^ Subcategory that represents acquired immunodeficiency other than AIDS. ^c^ Four patients with definite PML underwent autopsy after biopsy. ^d^ Subcategory that encompasses cases in which both FFPE and frozen samples were analyzed.

**Table 2:** Detection performance of CSF testing for JCPyV compared with pathological PML diagnosis of the brain tissue.

Performance	CSF Sampling ^a^	Number (%) of Patients			
		Standard PCR Assay		Ultrasensitive PCR Assay	
Sensitivity ^b^	Initial	10/25	(40)	34/40	(85)
	Initial and follow-up	n.a.	n.a.	38/40	(95)
False-positive rate ^c^	Initial	0/11	(0)	0/34	(0)
	Initial and follow-up	n.a.	n.a.	0/34	(0)
Positive predictive value ^d^	Initial	10/10	(100)	34/34	(100)
	Initial and follow-up	n.a.	n.a.	38/38	(100)
Specificity ^e^	Initial	11/11	(100)	34/34	(100)
	Initial and follow-up	n.a.	n.a.	34/34	(100)
False-negative rate ^f^	Initial	15/25	(60)	6/40	(15)
	Initial and follow-up	n.a.	n.a.	2/40	(5)
Negative predictive value ^g^	Initial	11/26	(42)	34/40	(85)
	Initial and follow-up	n.a.	n.a.	34/36	(94)

Data are presented as the number of individuals qualifying for CSF testing and pathological diagnosis of PML and the performance (%) of CSF testing in each category. CSF, cerebrospinal fluid; JCPyV, JC polyomavirus; PML, progressive multifocal leukoencephalopathy; n.a., not applicable. ^a^ For the ultrasensitive PCR assay, the data represent results for the initial test only as well as results including patients who underwent additional follow-up CSF testing (once: three patients; twice: one patient). ^b^ Proportion of CSF JCPyV-positive patients among definite PML cases. ^c^ Proportion of CSF JCPyV-positive patients among not PML cases. ^d^ Proportion of patients with definite PML among those who tested positive for CSF JCPyV. ^e^ Proportion of CSF JCPyV-negative patients among not PML cases. ^f^ Proportion of CSF JCPyV-negative patients among definite PML cases. ^g^ Proportion of patients with not PML among those who tested negative for CSF JCPyV.

## Data Availability

The analyzed datasets are available in the article or are available from the corresponding author upon reasonable request.

## Supplementary Materials

The following supporting information can be downloaded at https://www.mdpi.com/article/10.3390/v16121950/s1: Dataset S1: Nucleic acid extraction procedures for PCR testing of JCPyV; Dataset S2: Conditions for real-time PCR detection of JCPyV.
